# Exploring the wound healing potential of dietary nitrate in diabetic rat model

**DOI:** 10.3389/fphys.2024.1475375

**Published:** 2024-11-20

**Authors:** Xiaodan Hu, Haoyue Xu, Lingxue Bu, Jian Sun, Jiangzhi Deng, Kai Song, Lin Wang, Baoxing Pang

**Affiliations:** ^1^ Department of Oral and Maxillofacial Surgery, The Affiliated Hospital of Qingdao University, Qingdao University, Qingdao, China; ^2^ School of Stomatology of Qingdao University, Qingdao, China

**Keywords:** nitrate, nitric oxide, diabetic wounds, wound healing, angiogenesis

## Abstract

**Introduction:**

The wound healing in diabetes is hindered and prolonged due to long-term inflammation, oxidative stress damage, and angiogenesis disorders induced by high glucose status. The management of such difficult-to-treat wounds continues to pose a significant challenge in clinical treatment. Dietary nitrate, commonly found in greens such as beets and spinach, acts as a nutritional supplement and is metabolized in the body through the salivary nitrate-nitrite-NO pathway. This pathway plays a crucial role in various physiological functions, including enhancing blood flow and attenuating inflammation.

**Methods:**

In this study, we established a diabetic rat wound model. Forty-eight rats were randomly divided into six groups (n = 8): the Con group, the Con + Nitrate group, the STZ group, the STZ + NaCl group, the STZ + rhEGF group, and the STZ + Nitrate group. Skin wound healing was assessed on the day of surgery and on postoperative days 3, 7, 10, and 14. Specimens were taken on days 7 and 14 post-surgery for relevant tests.

**Results:**

We found that dietary nitrate could accelerate skin wound healing by promoting angiogenesis and increasing blood perfusion. Significantly, dietary nitrate also regulated glucose and lipid metabolism and exhibited anti-inflammatory and antioxidant properties.

**Discussion:**

These findings provide a novel theoretical basis for managing wounds in diabetic individuals, indicating the broad potential of dietary nitrate in future clinical applications.

## 1 Introduction

The healing process of diabetic wounds is complicated by factors such as hyperglycemia, vascular dysfunction, and chronic inflammation (Kalan et al., 2019; Liu W. et al., 2022; Wang et al., 2024). This poses significant challenges to patients’ quality of life and healthcare systems (Chen et al., 2023). Wound healing is a dynamic and synergistic phenomenon, necessitating a concerted interplay among an array of elements (Liu C. et al., 2022). Normal wound healing includes four stages: coagulation, inflammation, proliferation and remodeling (Hwang et al., 2022). However, the wound healing process in diabetic patients is often hindered and delayed, resulting in chronic hard-to-heal wounds (Kim et al., 2021). Among the many factors that affect wound repair, angiogenesis is particularly crucial (Yamashita et al., 2011). Inefficient diabetic wound healing is usually accompanied by local microcirculation disorders and reduced blood supply, affecting the supply of oxygen and nutrients to the wound, thereby delaying the healing process (Zha et al., 2022; Shao et al., 2023). Additionally, in patients with diabetes, high blood sugar levels can lead to abnormal or delayed inflammatory responses. This may inhibit the expression of anti-inflammatory factors and promote the production of a large number of inflammatory cells in the wound area, thus limiting wound closure (Bi et al., 2019). In addition, oxidative stress is a problem that cannot be ignored in diabetic wound healing (Han et al., 2024). High oxidative stress can lead to DNA damage, long-term inflammation, cell death, and subsequent tissue dysfunction, impeding wound healing (Shuvaev et al., 2011; Ma et al., 2022). Furthermore, the effect of glycolipid metabolism on diabetic wound healing is profound and complex. The nature of diabetes is due to an imbalance in glycolipid metabolism as a result of abnormalities in insulin utilization or secretion in the body (Wang D. et al., 2019; Xu et al., 2022). A prolonged hyperglycemic environment promotes non-enzymatic glycosylation reactions, resulting in the formation of advanced glycation end products (AGEs) that damage normal cellular function (Chen Y. et al., 2020). Dyslipidemia can lead to lipid peroxidation, resulting in impaired cell membrane stability and function (Yang et al., 2017). Collectively, these pathophysiological shifts adversely affect the wound healing process.

In recent times, avant-garde strategies for managing wounds associated with diabetes have included the utilization of biomaterials, stem cell therapy, and growth factor therapy (Wang et al., 2022). Biomaterials, notably bioscaffolds, serve a dual role in wound healing—providing structural support and promoting the repair of compromised tissues (Wei et al., 2023). Stem cell therapy has demonstrated significant promise in clinical settings, attributed to its tissue regeneration capabilities and facilitation of wound closure (Wang J. et al., 2019). Concurrently, growth factor therapy augments the healing process via the provision of biomolecules that encourage angiogenesis and cellular proliferation (Yan et al., 2018). Despite these advances, the economic burden and unresolved safety profiles of these methods pose challenges to their widespread adoption. Consequently, there is a compelling demand for the innovation of a straightforward and economically viable wound care modality that can mitigate costs and circumvent the emergence of drug resistance.

Nitrate, a natural component of the human diet, is abundantly found in green vegetables such as spinach, lettuce, and beets (Zhu et al., 2021). Nitrate in food is rapidly absorbed after entering the digestive tract and is converted to nitrite by oral bacteria. In the acidic stomach, it is further metabolized to produce nitric oxide (NO), maintaining the nitrate-nitrite-NO homeostasis in the body (Montenegro et al., 2016). Unlike NO synthase, the nitrate-nitrite-NO pathway functions independently of oxygen and L-arginine (Lundberg et al., 2009). This pathway is capable of operating in hypoxia and disease states, thus providing better prevention against cardiovascular diseases, ischemic diseases, and inflammatory diseases (Lundberg et al., 2008).

Increasing evidences suggest that beneficial effects of dietary nitrate may be mediated through the promotion of the nitrate-nitrite-nitric oxide pathway, thereby activating the multitude of physiological effects of nitric oxide. Numerous studies have demonstrated that dietary nitrate supplementation reduces oxygen consumption during exercise and increases athletes’ tolerance to high-intensity exercise (Husmann et al., 2019). Dietary nitrate has been posited to augment the chemosensitivity of oral squamous cell carcinoma, potentially through the amelioration of NO homeostasis within hypoxic tumor environments (Feng Y. et al., 2021). Emerging research suggests that dietary nitrate may represent a safe and effective therapeutic strategy for the management of radiation-induced xerostomia (Feng X. et al., 2021). In addition, inorganic nitrates have been shown to counteract NO deficiency associated with endothelial dysfunction, consequently attenuating the inflammatory activity of macrophages within atherosclerotic lesions (Khambata et al., 2017). In the context of ischemia-reperfusion-induced hepatic oxidative stress injury, nitrate supplementation has been demonstrated to mitigate hepatic oxidative stress. This is achieved through the upregulation of nuclear factor erythroid 2-related factor 2 (NRF2)-associated molecules, alongside an increase in the activities of antioxidant enzymes (Li et al., 2021). Dietary nitrate facilitates the nitrate-nitrite-NO pathway, thus offering partial compensation for deficits in endogenous NO synthesis. This action is pivotal in rectifying insulin signaling pathways and mitigating the effects of diabetes (Goh et al., 2019). Studies have shown that inorganic nitrate modulates lipid metabolism and improves glucose homeostasis in endothelial nitric oxide synthase (eNOS)-deficient mice (Hezel et al., 2015). Additionally, inorganic nitrate mitigated obesity that was induced by a high-fat diet in murine models and optimized glucose and lipid metabolic processes. This beneficial effect is attributed to the activation of the NO signaling pathway and the consequent modulation of intestinal microbiota composition (Ma et al., 2020).

The main characteristics of diabetic wounds include reduced angiogenesis and impaired microcirculation; thus, increasing blood flow and microcirculation within the wound is essential for facilitating the healing process of diabetic wounds. Some studies have found that dietary nitrate consumption not only mitigates blood pressure but also enhances vascular function (Raubenheimer et al., 2019). Research examining dietary nitrate’s influence on cerebral circulation and cognitive health revealed that nitrates boosted cerebral perfusion amidst visual-task stimulation when combined with medication (Gonçalves et al., 2024). Moreover, nitrate augments gastric mucosal blood flow (Björne et al., 2004), offering protection against mucosal injury induced by nonsteroidal anti-inflammatory drugs (NSAIDs) (Lanas, 2008). Our team’s earlier investigation discovered that dietary nitrate precluded skin flap damage through increased blood perfusion (Cui et al., 2019). Additionally, our research into the recuperation of infected cutaneous wounds in mice indicated that nitrate supplementation intensified microvessel density in the wound region, thus expediting the healing trajectory (Hu et al., 2023).

**FIGURE 1:**
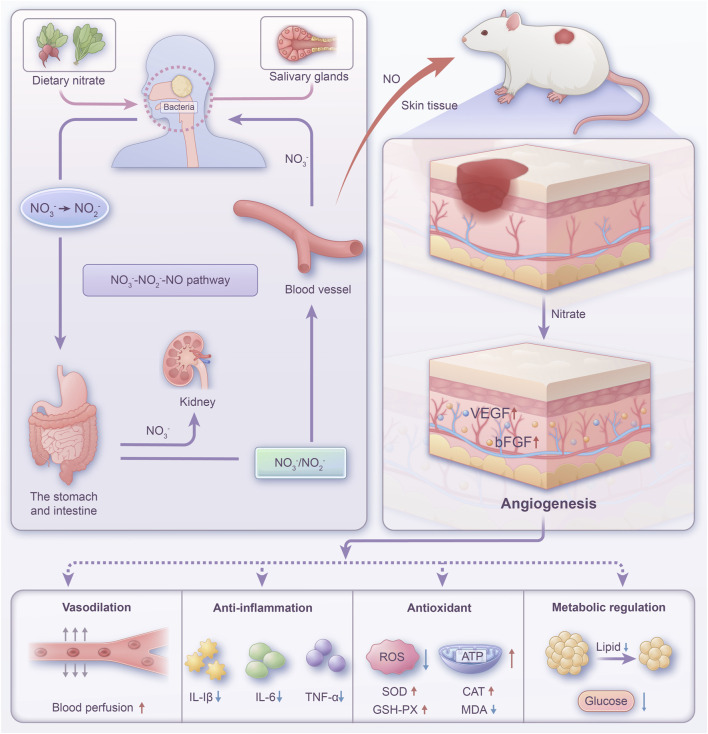
Schematic diagram of the pathway of nitrate circulation *in vivo* and the mechanisms of wound healing.

Here, we established a diabetic skin wound model in rats to elucidate the impact of dietary nitrate through the nitrate-nitrite-NO pathway on the healing of diabetic wounds. Figure 1 showed the schematic diagram of the pathway of nitrate circulation *in vivo* and the mechanisms of wound healing. Our research provided evidence that dietary nitrate modulated angiogenesis and ameliorated blood perfusion within wound tissues. Furthermore, supplementation with nitrate appeared to correct anomalies in glycolipid metabolism and displayed both anti-inflammatory and antioxidant qualities. These findings suggest that consuming nitrate-enriched green vegetables offers notable advantages in the treatment of diabetic wounds, thereby highlighting a potential clinical application.

## 2 Materials and methods

### 2.1 Animals and design

Male Sprague-Dawley rats (180–200 g) were purchased from Pengyue Laboratory Animal Breeding Center (Jinan, China). All animal experiments were approved by the Committee of Animal Care and Welfare in the Affiliated Hospital of Qingdao University (Protocol No. AHQU20190107A). Before surgery, the animals were housed for at least 7 days in a ventilated, temperature-controlled room and were allowed to drink and eat freely. After rats were fed a high-fat diet (HFD) for 8 weeks, a diabetes model was established by administering streptozotocin (STZ, 40 mg/kg, intraperitoneal injection, i. p.) dissolved in sodium citrate buffer (pH 4.5). Random blood glucose levels were measured 1 week after STZ injection, with rats having blood glucose levels above 16.67 mmol/L being selected for subsequent experiments. Control animals were given an equivalent volume of sodium citrate buffer (pH 4.5). Two weeks post-STZ induction, the rats were anesthetized with sodium pentobarbital (50 mg/kg, intraperitoneal injection, i. p.) and a full-thickness wound of approximately 1.5 cm in diameter was created on their backs. Forty-eight rats were randomly divided into six groups (n = 8): the Con group (normal water, non-diabetic), the Con + Nitrate group (water containing 2 mM NaNO3, non-diabetic), and the STZ group (normal water, diabetic), the STZ + NaCl group (water containing 2 mM NaCl, diabetic), the STZ + rhEGF group [normal water, diabetic, recombinant human epidermal growth factor (rhEGF)], and the STZ + Nitrate group (water containing 2 mM NaNO3, diabetic). The Con + Nitrate group and the STZ + Nitrate group began to consume nitrate-containing water 1 week prior to surgery. Skin wound healing was assessed on the day of surgery and on postoperative days 3, 7, 10, and 14. Rats were anesthetized with 3% isoflurane (RWD, China) mixture with oxygen, photographs of the wounds were taken with a digital camera, and the area of the wounds was quantified using ImageJ software. Specimens were taken on days 7 and 14 post-surgery for relevant tests.

### 2.2 Blood perfusion measurement

A Moor VMS-LDF (Moor Instrument, United Kingdom) was utilized to monitor blood flow variations in the wound skin. Laser Doppler Flowmetry (LDF) measurements were conducted in accordance with the manufacturer’s specifications. Each wound was subdivided into nine partitions by both horizontal and vertical lines. Random sampling was performed using a computer-generated random number method. The final blood perfusion of the wound was determined by calculating the average of at least six random perfusion unit values within each wound. The blood perfusion change rate was calculated on days 7 and 14 post-operation to track the changes in blood flow (blood perfusion change rate = blood perfusion value measured on day 7 or 14/blood perfusion value measured pre-operation × 100).

### 2.3 Determination of nitrate, nitrite content in the serum and tissue

Serum and skin tissue were obtained and homogenized to collect the supernatant after operation. The concentrations of nitrate and nitrite in serum were detected by Total Nitric Oxide and Nitrate/Nitrite Parameter Assay Kit (KGE001, R&D, United States) according to the manufacturer’s instructions.

### 2.4 Histological and immunohistochemical analysis

Rats were euthanized with sodium pentobarbital (130 mg/kg, i. p.) at days 7 and 14 post-operation, and the skin tissue encompassing the wound was excised for histological analysis. The specimens were fixed in 4% paraformaldehyde, subsequently embedded in paraffin, and sectioned into 5-μm slices. For the assessment of re-epithelialization and collagen synthesis, the sections underwent staining with H&E and Masson’s trichrome, respectively. The quantification of collagen content was performed using ImageJ software. Furthermore, the sections underwent both immunohistochemical and immunofluorescent staining for CD31 (Abcam, AB28364), VEGF (R&D Systems, AF564), bFGF (R&D Systems, AF-233-NA), α-SMA (Abcam, AB32575), IL-6 (Affinity, DF6087), IL-1β (Affinity, AF5103), TNF-α (Affinity, AF7014) and caspase 3 (BD Pharmingen, 559565).

### 2.5 Evaluation of ATP and oxidative stress biomarkers in homogenate

The levels of ATP, ROS, CAT, GSH-Px, SOD, and MDA in tissue homogenate were evaluated using commercial kits (Nanjing Jiancheng Bioengineering Institute, Nanjing, China) according to the manufacturers’ instructions.

### 2.6 Glucose tolerance and insulin resistance analysis

Selected rats were subjected to an intraperitoneal glucose tolerance test (IPGTT). Following a 12-h fasting period, D-glucose (1.5 g/kg, SUNNCELL, SNSP-001) was administered intraperitoneally to the rats. Subsequent to administration, blood samples were drawn from the tail vein at designated time intervals, and glucose levels were monitored using a glucometer (Roche, Germany). In addition, an insulin tolerance test (ITT) was conducted to evaluate insulin sensitivity. After fasting for 6 h, insulin (0.75 UI/kg, Novo Nordisk) was injected intraperitoneally into the rats, and blood glucose levels were measured at the corresponding time points as previously mentioned. The areas under the curve (AUC) were calculated for IPGTT and ITT.

### 2.7 Biochemical analysis

At the conclusion of the experiment, the rats underwent a 12-h fasting period prior to the collection of blood samples. Blood was drawn from the heart and collected into coagulation tubes, then centrifuged at 3,000 *g* for 15 min to separate the serum. Serum levels of total TC, TG, HDL, LDL, and blood glucose were measured using an AU5400TM automatic biochemical analyzer (Olympus Optical, Japan). Additionally, insulin levels were quantified with a commercial ELISA kit (Invitrogen, United States).

### 2.8 Statistical analysis

All data were expressed as mean ± standard deviation (SD) and analyzed by GraphPad Prism 9.0. One-way ANOVA followed by Tukey’s test or two-way ANOVA test followed by Bonferroni’s test were utilized for the evaluation of the significant difference, setting the level at *P* < 0.05 as statistical significance.

## 3 Results

### 3.1 Nitrate therapy accelerated the wound closure in diabetic rats

**FIGURE 2:**
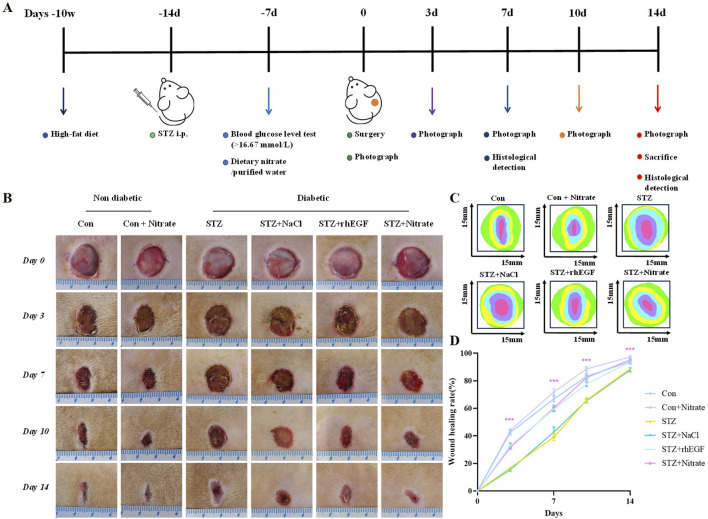
Schematic diagram of wound healing in diabetic rats. **(A)** The animal experimental design. **(B)** Representative images of wound healing in each group at day 0, 3, 7 and 14. **(C)** Traces of wound closure area for 14 days. **(D)** Wound healing rate in each group at day 0, 3, 7 and 14, n = 4.^*^ *P* < 0.05, ^**^ *P* < 0.01, ^***^ *P* < 0.001.

To evaluate the effect of dietary nitrate on diabetic wound healing, we allowed rats to have a high-fat diet for 8 weeks followed by intraperitoneal injection of streptozotocin (STZ) to induce a diabetic model. Forty-eight rats were randomly divided into six groups (n = 8): the Con group, the Con + Nitrate group, the STZ group, the STZ + NaCl group, the STZ + rhEGF group, and the STZ + Nitrate group. The Con + Nitrate group and the STZ + Nitrate group began drinking nitrate-containing water 1 week before surgery. Skin wound healing was assessed on the day of surgery, as well as on days 3, 7, and 14 post-surgery. The rats were anesthetized using gas anesthesia, photographs of the wounds were captured with a digital camera, and the wound area was quantified using ImageJ software. The diabetic wound model procedures and timescales were shown in Figure 2A. The body weight of the diabetic modeled rats gradually decreased over a period of 14 days and blood glucose was maintained at a high level (Supplementary Figure S1).

As shown in Figures 2B–D, our initial experiments confirmed that nitrate supplementation played a positive role in wound healing in non-diabetic rats. To further investigated the effect of dietary nitrates on refractory diabetic wounds, we compared the effect of nitrate with rhEGF treatment. The results showed that wound healing was significantly slower in the STZ group than in normal rats. In STZ-induced diabetic wounds, the wound healing rate in the STZ + NaCl group was similar to that in the STZ group. On the contrary, the wound healing effect of the dietary nitrate group was comparable to that of the STZ + rhEGF group, and the healing rate was faster than that of diabetic rats, indicating that dietary nitrate could effectively promote the healing of diabetic skin wounds.

### 3.2 Dietary nitrate promoted re-epithelialization and collagen deposition

**FIGURE 3:**
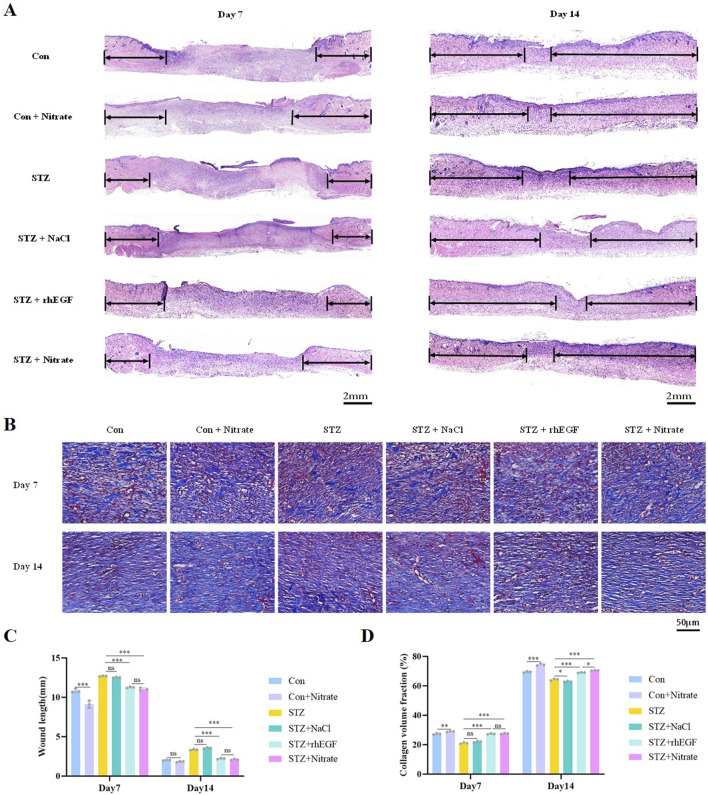
Dietary nitrate promoted re-epithelialization and collagen deposition. **(A, C)** H&E staining analysis was performed on wound sections at day 7 and 14 post-wounding, with arrows indicating areas of epithelialization. Scale bars = 2 mm. **(B, D)** Masson’s trichrome staining was conducted at day 7 and 14 post-wounding to assess collagen volume fraction. Scale bars = 50 μm. n = 3, ^*^ *P* < 0.05, ^**^ *P* < 0.01, ^***^ *P* < 0.001.

Nitrate supplementation positively influenced wound re-epithelialization and collagen deposition in both diabetic and non-diabetic rats. Effective re-epithelialization plays a crucial role in successful skin wound healing. When skin sustains damage, adjacent healthy epithelial cells initiate proliferation and migration towards the wound’s center, contributing to the formation of a new epithelial layer that seals the wound (Lee et al., 2022). This regenerative process reestablishes the skin’s integrity and barrier function, thereby safeguarding against the incursion of external microorganisms and foreign matter. The groups treated with STZ + Nitrate and STZ + rhEGF demonstrated enhanced re-epithelialization and accelerated wound healing, with histological analysis via hematoxylin-eosin (H&E) staining revealing a greater number of neo-epithelial structures compared to the STZ monotherapy group (Figures 3A, C). Conversely, the STZ + NaCl group showed re-epithelialization rates similar to the STZ group, both showed a slightly lower trend (Figures 3A, C).

Collagen synthesis and deposition are essential for wound healing, not only providing mechanical support, but also playing a crucial role in promoting cell adhesion, proliferation and differentiation (Zhao et al., 2017). Masson’s trichrome staining demonstrated that nitrate-treated diabetic wounds could enhance collagen production to a level comparable to the STZ + rhEGF group, even surpassed the STZ + rhEGF group at day 14 (Figures 3B, D). In the STZ + Nitrate group, collagen deposition was more extensive, arranged more orderly, and had thicker collagen fiber bundles (Figures 3B, D). These findings suggest that dietary nitrate significantly enhances wound repair by accelerating re-epithelialization and collagen deposition.

### 3.3 Nitrate supplementation promoted angiogenesis and increases blood perfusion

**FIGURE 4:**
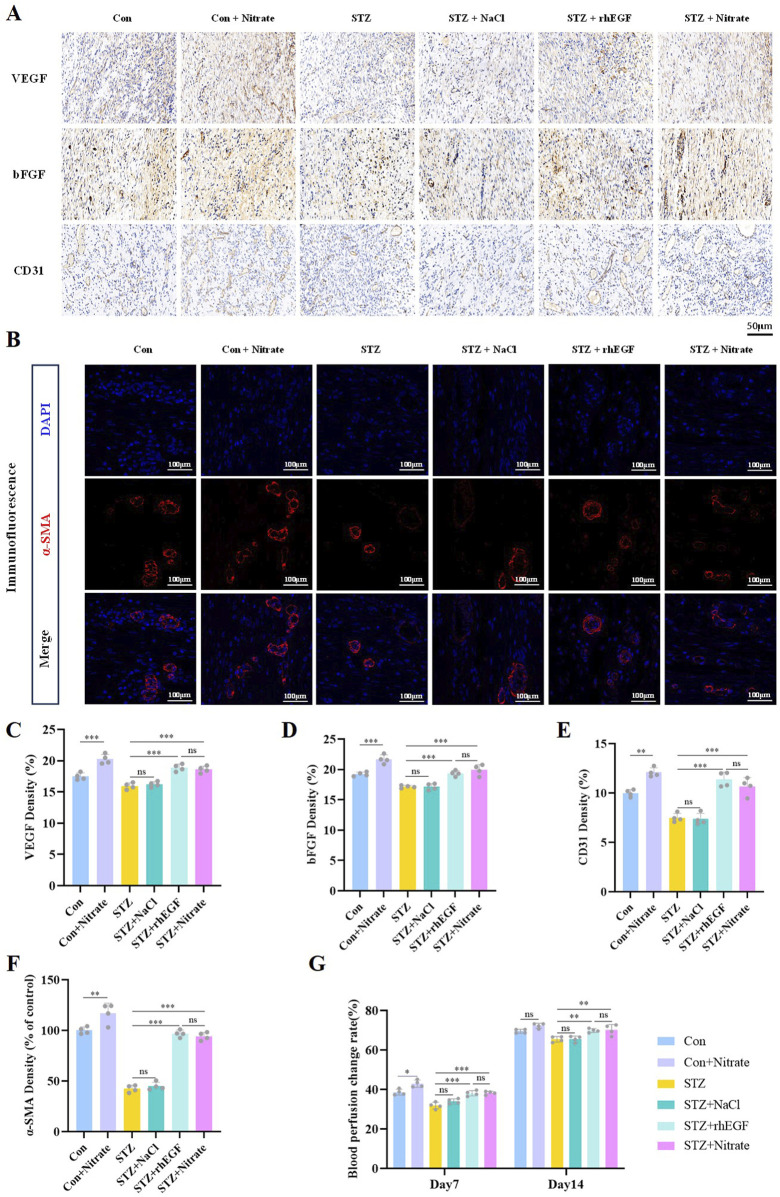
Nitrate supplementation accelerated angiogenesis and increases blood perfusion. **(A, C–E)** Immunohistochemical staining of VEGF, bFGF and CD31 on day 14 after wound healing and their quantitative results. Scale bar = 50 μm. **(B, F)** Immunofluorescence staining of α-SMA at day 14 post-wounding and its quantitative results. Scale bars = 100 μm. **(G)** Blood perfusion change rates at day 7 and 14 post-wounding. n = 4, ^*^ *P* < 0.05, ^**^ *P* < 0.01, ^***^ *P* < 0.001.

Neovascularization was crucial for effective wound healing, serving as a vital source of oxygen and nutrients. Vascular endothelial growth factor (VEGF) and basic fibroblast growth factor (bFGF) are two critical growth factors integral to the induction of angiogenesis. VEGF stands out as one of the most potent angiogenic agents, accelerating the proliferation and migration of vascular endothelial cells, ultimately leading to neovascularization primarily through its interaction with VEGF receptors on endothelial cell surfaces (Chen J. et al., 2020). Meanwhile, bFGF acts as a vital angiogenic modulator, involved in the degradation and remodeling of the basement membrane as well as in endothelial cell proliferation and migration (Presta et al., 2005). This study’s findings demonstrate that both dietary nitrate supplementation and topical rhEGF treatment significantly elevate VEGF and bFGF expression in diabetic rats (Figures 4A, C, D and Supplementary Figure S2).

CD31, also known as platelet endothelial cell adhesion molecule 1, is commonly employed as a biomarker for vascular endothelial cells. Quantitative analysis of CD31 expression in tissue samples enables an assessment of vascular density, thereby providing insights into the extent of neovascularization. In the continuum of neovascularization, the expression of α-SMA (α-smooth muscle actin) notably augments within the vessel wall. This denotes a pivotal transition from an incipient endothelial cell lumen toward a structurally mature vessel (Saigusa et al., 2015). Consequently, measuring the expression levels of α-SMA in tissues serves as an indicator of neovascular maturity. Data from the current investigation indicate that dietary nitrate somewhat increased vascular density within the wound regions of both normoglycemic and diabetic rats. Notably, the number of vessels in the STZ + nitrate group was comparable to that of the STZ + rhEGF group, but higher than that of the STZ group (Figures 4A, B, E, F and Supplementary Figure S2). These findings present evidence that nitrate treatment can elevate microvessel density and fosters a robust vascular network within the wound matrix.

The presence of new blood vessels resulted in enhanced blood flow, leading to improved blood perfusion in the wound area. On days 7 and 14 post-surgery, the blood perfusion levels in the STZ + Nitrate group and STZ + rhEGF group were consistently high, whereas a decrease in blood perfusion was observed in the STZ group and STZ + NaCl group (Figure 4G).

### 3.4 Nitrate intake attenuated inflammatory response and inhibited apoptosis

**FIGURE 5:**
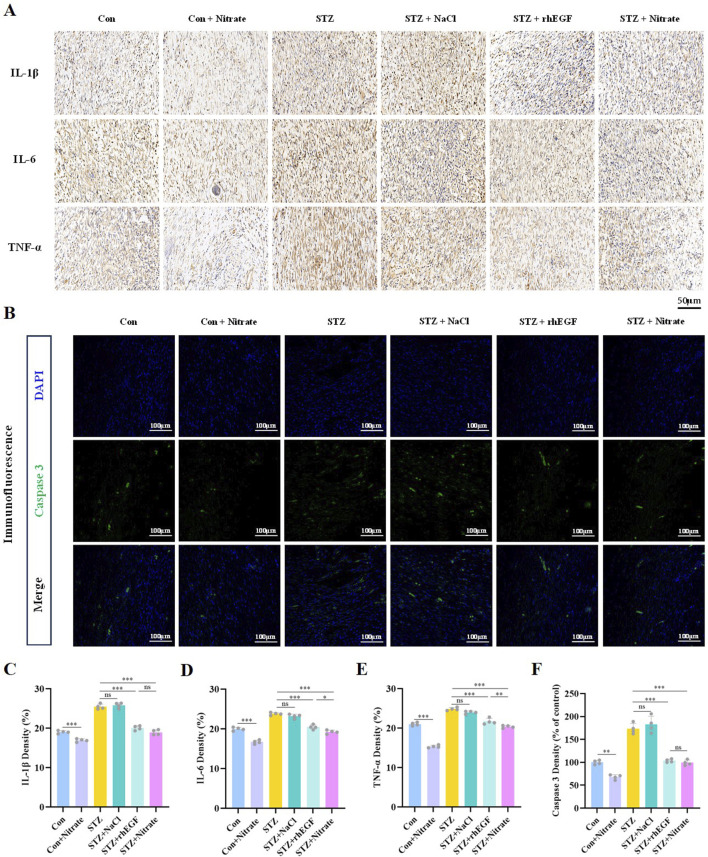
Nitrate intake attenuated the inflammatory response and inhibited apoptosis. **(A, C–E)** Immunohistochemical staining of IL-1β, IL-α, and TNF-α and their quantitative results on day 14 after wound healing. Scale bar = 50 μm. **(B, F)** Immunofluorescence staining of caspase 3 at day 14 post-wounding and its quantitative results. Scale bars = 100 μm. n = 4, ^*^ *P* < 0.05, ^**^ *P* < 0.01, ^***^ *P* < 0.001.

In the context of diabetes, impaired wound healing is often attributed to the excessive recruitment and prolonged presence of pro-inflammatory cells and mediators. To elucidate the potential anti-inflammatory effects of dietary nitrate, this study employed immunohistochemical staining techniques to quantify the expression of prototypical inflammatory cytokines within wound tissues during the healing process. Interleukin 1 beta (IL-1β), Interleukin 6 (IL-6), and Tumor Necrosis Factor alpha (TNF-α) are recognized as pivotal regulators within the immune system, frequently acting in a synergistic manner to modulate inflammatory and immune responses. A notable positive feedback loop exists among these modulators: the inflammatory reaction triggered by IL-1β and TNF-α further incites the secretion of IL-6, which in turn promotes the generation of more IL-1β and TNF-α (Li et al., 2016), thus exacerbating and sustaining the inflammatory state. Observations from this investigation revealed a significant diminution in the levels of inflammatory markers such as IL-1β, IL-6, and TNF-α in the STZ + Nitrate group following nitrate administration, in stark contrast to the sustained high-level inflammation observed in both the STZ and the STZ + NaCl groups (Figures 5A, C–E and Supplementary Figure S3). These findings imply that dietary nitrate may possess a meaningful anti-inflammatory capacity, potentially expediting the wound healing process.

The process of wound healing extends beyond mere cellular growth and differentiation. It encompasses the purging of surplus, undesired, or impaired cells conducted through apoptosis. The activation of this apoptotic pathway, crucial for discarding irreversibly damaged cells, hinges on the vital enzyme caspase 3, which triggers the apoptosis program (Lee et al., 2016). The expression levels of caspase 3 serve as a predictive indicator of the presence of damaged cells within the wound. Immunofluorescence staining presented a decline in caspase 3 activity within wound tissues subjected to nitrate supplementation (Figures 5B, F), intimating that dietary nitrate might function to impede apoptosis, thereby fostering more efficient wound healing.

### 3.5 Dietary nitrate increased nitrate, nitrite levels and alleviated oxidative stress

**FIGURE 6:**
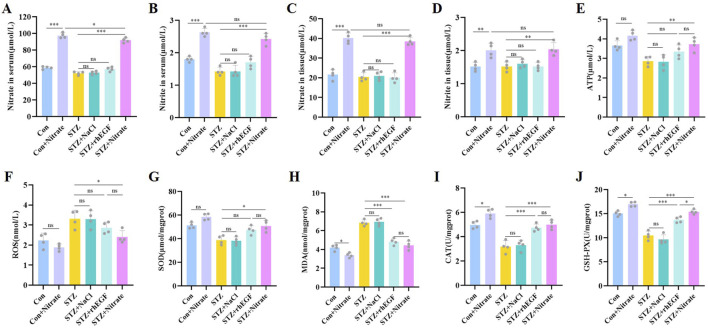
Effects of dietary nitrate on nitrate, nitrite and oxidative stress *in vivo*. Serum levels of nitrate **(A)**, nitrite **(B)** on day 14 after wound healing. Tissue levels of nitrate **(C)**, nitrite **(D)** on day 14 after wound healing. Tissue levels of ATP **(E)**, ROS **(F)**, SOD **(G)**, MDA **(H)**, CAT **(I)**, GSH-PX **(J)** on day 14 after wound healing. n = 4, ^*^ *P* < 0.05, ^**^ *P* < 0.01, ^***^ *P* < 0.001.

Upon completion of the experimental protocol, the rats underwent humane euthanasia, followed by the collection and analysis of blood specimens. Study findings revealed a marked elevation in the levels of nitrate and nitrite in both serum and tissue (Figures 6A–D), consequent to the ingestion of dietary nitrate. This outcome is likely indicative of the enhanced efficiency of the nitrate-nitrite-NO pathway, facilitated by nitrate supplementation, which augments nitrite and NO synthesis. Hence, dietary nitrate proves to be a potent means of generating NO and reactive nitrogen species, suggesting that incorporating nitrate in the diet could modulate nitrate-nitrite-NO pathway within the organism, thereby expediting the wound healing process.

Mitochondria serve as the cellular powerhouses, chiefly tasked with adenosine triphosphate (ATP) generation, a molecule pivotal for energy currency. ATP is integral to support substantial cellular functions such as proliferation, migration, differentiation, and matrix construction during the wound healing process. Our investigation revealed an upswing in ATP levels in the wound tissues of diabetic rat post nitrate supplementation, whereas in the STZ and STZ + NaCl groups, ATP levels remained comparatively low (Figure 6E). This suggests that nitrate supplementation might enhance mitochondrial functionality and ATP synthesis. Additionally, lipid peroxidation, instigated by oxygen free radicals, leads to oxidative damage in wounds, typified by an upsurge in reactive oxygen species (ROS) which, in turn, result in cellular impairment and subsequent cell demise (Joshi et al., 2016), thereby hindering the healing process. Addressing this, bolstering the body’s antioxidant defenses is essential to expedite wound healing. Indicators such as the activities of superoxide dismutase (SOD), catalase (CAT), and glutathione peroxidase (GSH-Px), along with malondialdehyde (MDA) levels, are critical in appraising oxidative stress severity. Our data illustrated that ROS and MDA levels were substantially elevated, with concomitant declines in SOD, CAT, and GSH-Px activities in the skin tissues of diabetic rats with wounds—indicative of diminished antioxidative response and disrupted redox homeostasis (Figures 6F–J). Conversely, the STZ + Nitrate and STZ + rhEGF groups showed reduced ROS and MDA levels and significantly heightened activities of SOD, CAT, and GSH-Px (Figures 6F–J).

### 3.6 Nitrate therapy improved glucose tolerance and insulin resistance and modulated lipid metabolism

**FIGURE 7:**
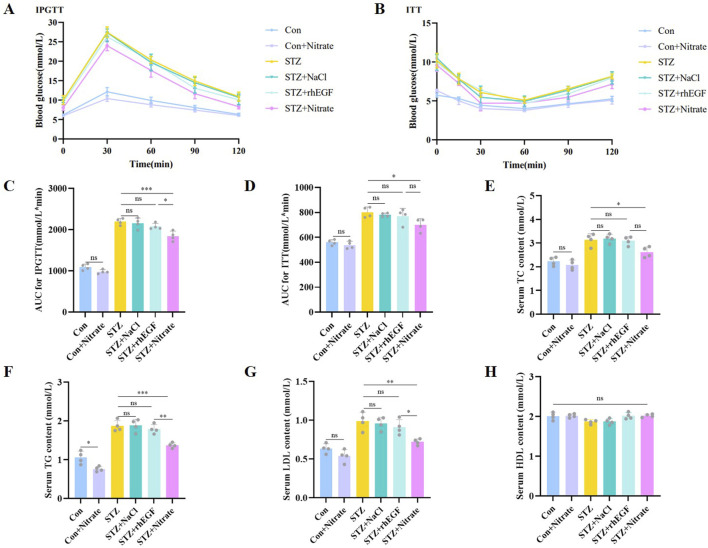
Dietary nitrate modulated glucolipid metabolism in diabetic rats. **(A)** Glucose changes in the IPGTTs. **(B)** Glucose changes in the ITTs. **(C)** AUC for IPGTTs were presented in the column graph. **(D)** AUC for ITTs were presented in the column graph. Blood lipid indicators including TC **(E)**, TG **(F)**, LDL **(G)** and HDL **(H)** were measured at the end of the experiment. n = 4, ^*^ *P* < 0.05, ^**^ *P* < 0.01, ^***^ *P* < 0.001.

In the intraperitoneal glucose tolerance test (IPGTT), diabetic rats demonstrated impaired glucose regulation relative to nondiabetic controls (Figures 7A, C). However, post-nitrate supplementation, the STZ + nitrate group exhibited a marked improvement in glucose regulation, also reflected by increased insulin sensitivity as observed in the insulin tolerance test (ITT) (Figures 7B, D). These results imply that nitrate supplementation may bolster glucose tolerance and insulin sensitivity, beneficially influencing wound healing.

Maintaining optimal lipid levels is essential for effective wound healing. Deviations–either elevations or reductions in lipid concentrations–can disrupt the wound’s blood supply and tissue repair mechanisms, consequently retarding the healing process. Biochemical serum analysis indicated that diabetic rats had heightened total cholesterol (TC), triglycerides (TG), and low-density lipoprotein cholesterol (LDL-C) compared to their healthy counterparts (Figures 7E–G). Notably, these levels were substantially reduced in the STZ + nitrate group, suggesting nitrate’s regulatory potential on lipid metabolism. Conversely, the STZ + NaCl and STZ + rhEGF groups mirrored the STZ group’s lipid profiles. It is important to mention that high-density lipoprotein (HDL) levels did not differ significantly amongst the diabetic cohorts (Figure 7H).

## 4 Discussion

The pathological progression of diabetic wound healing operates within a dauntingly complex milieu, characterized by hyperglycemia, hypoxic conditions, and oxidative stress. This study focused on an exploration of the effects exerted by dietary nitrate on wound healing in a diabetic rat model, alongside an elucidation of underlying mechanisms, aspiring to present a straightforward and efficacious approach to wound management. Our observations indicated that nitrate consumption led to a marked elevation in the concentrations of nitrate and nitrite within both the bloodstream and wound tissue. The data suggests that dietary nitrate intake fosters re-epithelialization of wounds, expedites the accumulation of collagen, and significantly hastens the wound healing trajectory. A pivotal discovery of our research was the identification of dietary nitrates’ role in promoting neoangiogenesis and improving compromised angiogenesis, thereby enhancing blood perfusion in the wound area. Concurrently, the intake of dietary nitrate was noted to diminish the inflammatory response and exert an inhibitory effect on cellular apoptosis. Furthermore, nitrate exerted a regulatory influence on mitochondrial functionality, which could potentially curtail the production of ROS and alleviate oxidative stress. Dietary nitrate also exhibited beneficial actions on metabolic dysfunctions, including heightened glucose tolerance, augmented insulin sensitivity, and the restoration of lipid metabolism equilibrium. Herein, we report, for the first instance, a positive correlation linking dietary nitrate to diabetic wound healing, postulating that the angiogenic capacity may significantly contribute to the mechanistic aspects of this process.

Nitrate is found mainly in a variety of green vegetables, especially beets and spinach. The intake of nitrates from these vegetable sources can comprise up to 80% of the overall daily dietary consumption (Bondonno et al., 2021). Nitrate consumed through dietary means are distributed across the body, including the bloodstream, saliva, and various tissues. While a significant proportion of nitrate is ultimately eliminated via urine, approximately 25% is actively absorbed and retained by the salivary glands (Lundberg, 2012). Upon the consumption of nitrate-rich foods, oral bacteria facilitate the reduction of nitrate to nitrite. In the acidic environment of the stomach, nitrite spontaneously breaks down to form NO and other nitrogen oxides that are biologically active. These compounds are instrumental in the regulation of critical physiological functions. Subsequently nitrate and most of the remaining nitrite are absorbed by the small intestine into the circulation, where they are converted to biologically active NO to play a role in the blood and tissues under pathological and hypoxic conditions. This process is known as the nitrate-nitrite-NO pathway. The nitrate-nitrite-NO pathway plays a crucial role in mediating a range of NO-mimetic effects in the body. Researches have indicated that nitrate can reduce blood pressure through vasodilation (Kapil et al., 2015), regulate lipid metabolism aiding in obesity management (Ma et al., 2020), and enhance athletic performance by boosting the efficiency of oxygen utilization in muscles (Martínez-Noguera et al., 2022). Furthermore, dietary nitrates are implicated in the modulation of various cellular signaling cascades, including the MAPK and PI3K-Akt pathways, glutathione metabolism, and cell cycle regulation (Ene et al., 2023). These pathways are integral to cellular metabolism and have significant implications in the progression of various diseases.

Contrary to the earlier unsubstantiated perspective that nitrate is detrimental to health, a growing body of research is substantiating the positive impact of nitrate and its downstream products on human physiology. Relationship linking nitrate and nitrite intake to cancer risk has generated considerable debate. Apprehensions associated with such health risks often stem from the consumption of processed meats or ingestion of drinking water containing excessive nitrate and nitrite levels. Methemoglobinemia, a condition linked to high nitrate content in drinking water, is predominantly observed in infants and predisposes them to asphyxiation (Devereux et al., 2021). As a preventive measure, nitrate concentrations in drinking water are regulated to safeguard newborns from this condition. Frequent consumption of processed meats has been implicated in elevated cancer risk, particularly colorectal cancer (Bashir et al., 2020). However, a definitive correlation between red and processed meat consumption and gastrointestinal cancers remains elusive due to the current scarcity of conclusive evidence. Carcinogenicity research should pay particular attention to the production of N-nitroso compounds (NOCs) (Kotopoulou et al., 2022). At present, there’s no definitive evidence to associate the intake of nitrates and nitrites from fruits and vegetables with carcinogenicity. On the contrary, some studies have shown that taking nitrate extends the lifespan of fruit flies (Carvalho et al., 2021). Numerous studies corroborate the health advantages of nitrates and nitrites found in fruits and vegetables, especially in terms of bolstering cardiovascular and metabolic health.

In recent years, nitrate-rich greens such as beets, specifically beetroot juice, have emerged as focal points in scientific research due to their substantial nitrate composition. This interest is largely fueled by the potential health impact of dietary nitrates, particularly concerning human cardiovascular health and athletic performance. The ingestion of beets or their juice significantly augments one’s dietary nitrate consumption (Porcelli et al., 2016). Empirical evidence suggests that the consumption of beetroot can bolster athletic performance, which has propelled its popularity as a natural supplement among athletes and those devoted to fitness (Jagim et al., 2019). Furthermore, the broad spectrum of antioxidants found in beets, such as polyphenols and vitamins, exhibit anti-inflammatory and antioxidative properties (Nowacka et al., 2019), mitigating oxidative stress and offering protection against various chronic diseases. A substantial body of research has also dedicated attention to the nitrate content in beets for its cardiovascular benefits; these nitrates are metabolized into nitric oxide, a compound instrumental in reducing hypertension and enhancing vascular function (Bondonno et al., 2021). Owing to their considerable nutritional profile and health benefits, beets hold a valuable role in both wholesome and plant-based diets, and they show promise as an exemplary natural source for nitrate supplementation in the future.

In summary, this animal study has demonstrated that dietary nitrate can expedite the healing of diabetic wounds by fostering angiogenesis. Additionally, it offers an initial exploration of the potential mechanisms involved. Consequently, dietary nitrates hold promise as an innovative therapeutic approach for clinical applications.

## Data Availability

The raw data supporting the conclusions of this article will be made available by the authors, without undue reservation.
