# Supplementary material for: Exploring the wound healing potential of dietary nitrate in diabetic rat model
Source: Front Physiol. 2024 Nov 20;15:1475375. doi: 10.3389/fphys.2024.1475375 (PMC11614883; doi:10.3389/fphys.2024.1475375)
Supplement: Supplementary file 1 [file Table1.docx]

Supplementary Material


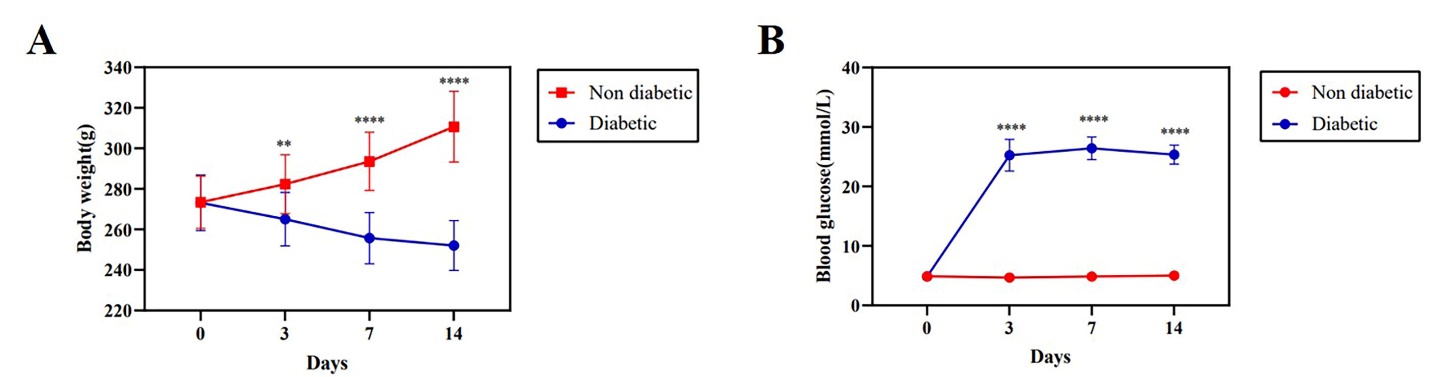


**Supplementary Figure S1.** Schematic illustration of body weight and blood glucose changed after modeling of diabetes in rats. (**A**) Body weight changed in rats modeled for diabetes. (**B**) Blood glucose changed in rats modeled for diabetes. Non diabetic (n=16), diabetic (n=32), ^*^*P* < 0.05, ^**^*P* < 0.01, ^***^*P* < 0.001, ^****^*P* < 0.0001.


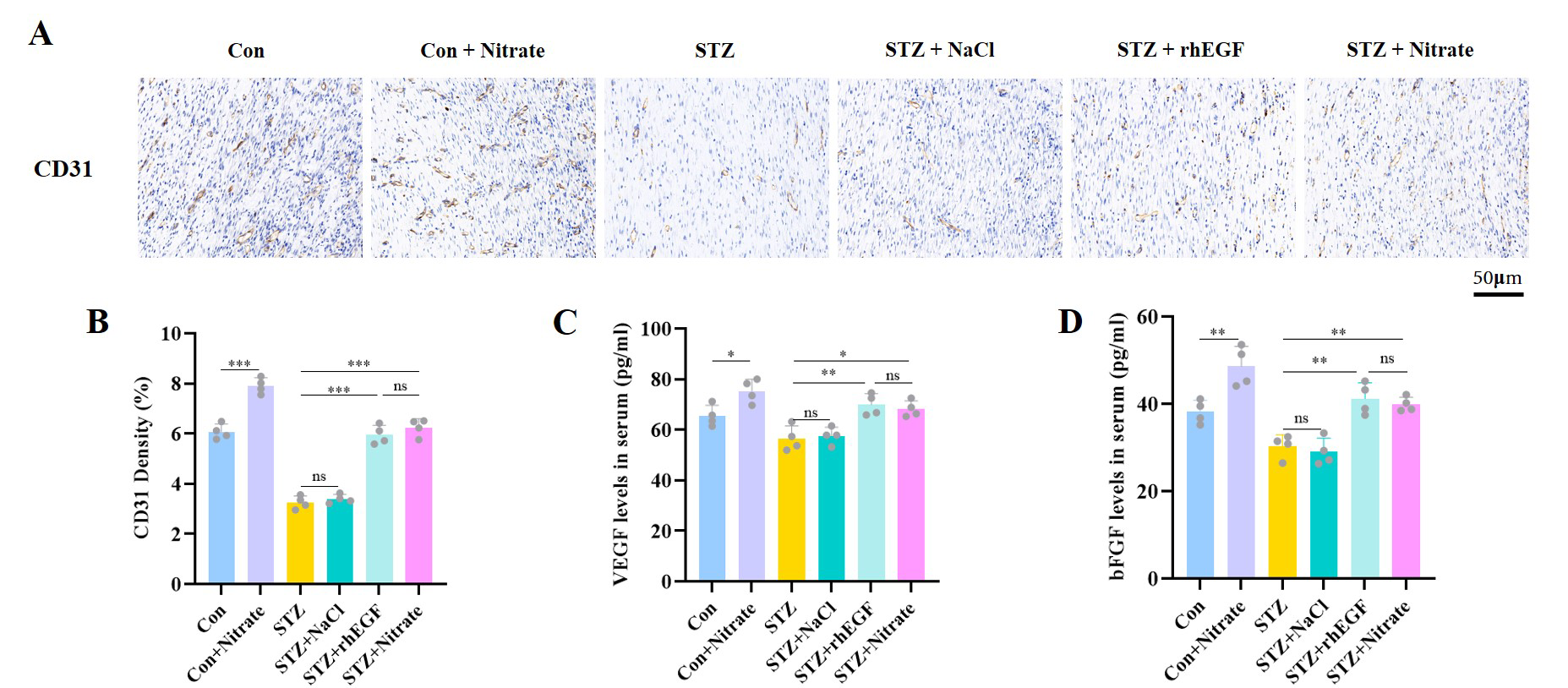


**Supplementary Figure S2.** Nitrate supplementation accelerated angiogenesis. (**A** and **B**) Immunohistochemical staining of CD31 on day 7 after wound healing and it quantitative results. Scale bar = 50 μm. (**C**) Serum levels of VEGF on day 14 post-operatively. (**D**) Serum levels of bFGF on day 14 post-operatively. n=4, ^*^*P* < 0.05, ^**^*P* < 0.01, ^***^*P* < 0.001.


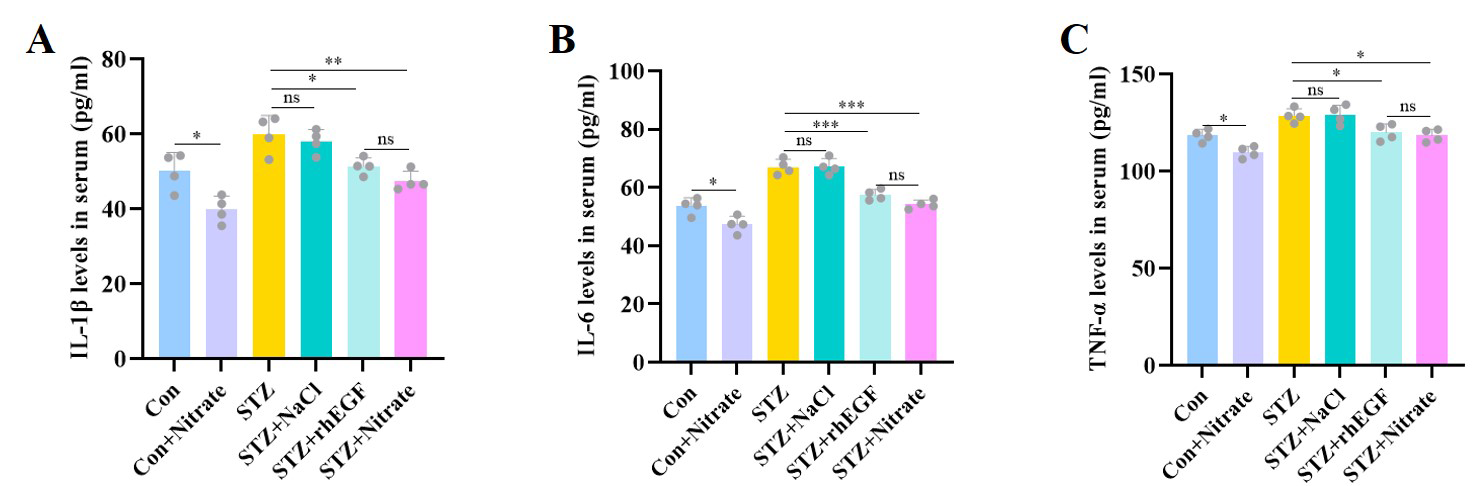


**Supplementary Figure S3.** Dietary nitrate reduced serum inflammatory factor levels. (**A**) Serum levels of IL-1β on day 14 post-operatively. (**B**) Serum levels of IL-6 on day 14 post-operatively. (**C**) Serum levels of TNF-α on day 14 post-operatively. n=4, ^*^*P* < 0.05, ^**^*P* < 0.01, ^***^*P* < 0.001.
